# Impact of Ergot Alkaloids on Female Reproduction in Domestic Livestock Species

**DOI:** 10.3390/toxins11060364

**Published:** 2019-06-21

**Authors:** Rebecca K. Poole, Daniel H. Poole

**Affiliations:** Department of Animal Science, North Carolina State University, Raleigh, NC 27607, USA; rkpoole@ncsu.edu

**Keywords:** ergot alkaloids, livestock, reproduction

## Abstract

Fescue toxicosis is a multifaceted syndrome that elicits many negative effects on livestock consuming ergot alkaloids produced by endophyte-infected tall fescue. The economic losses associated with fescue toxicosis are primarily due to reproductive failure including altered cyclicity, suppressed hormone secretion, reduced pregnancy rates, agalactia, and reduced offspring birth weights. For decades, a multitude of research has investigated the physiological and cellular mechanisms of these reproductive failures associated with fescue toxicosis. This review will summarize the various effects of ergot alkaloids on female reproduction in grazing livestock species.

## 1. Introduction

Ergot alkaloid mycotoxins were first identified in *Claviceps*, a parasitic fungus that infects many grasses and grains [1]. These mycotoxins are produced by a variety of fungi, including *Neotyphodium* and *Epichloë*, and classified as the tall fescue endophyte [1,2,3]. Specifically, *Epichloë coenophiala* is recognized as the endophyte that shares a symbiotic relationship with Kentucky (KY)-31 tall fescue (*Lolium arundinaceum* [Schreb.] Darbysh; [4]). Chronic consumption of *Epichloë coenophiala*-produced ergot alkaloids in grazing livestock results in a syndrome known as fescue toxicosis [5]. With KY-31 being estimated to be grown on 35 million acres of land in the Southeast to Midwest U.S. regions and that 90% of these pastures are infected with the *Epichloë* endophyte [6,7,8], it is speculated that fescue toxicosis contributes to over $2 billion in annual economic loss to the U.S. livestock industries [9]. Detrimental signs of this syndrome include reduced intake, weight gain, circulating prolactin concentrations, reproductive performance, milk production, and hyperthermia [10]. Many of these signs of fescue toxicosis can be attributed to the structural similarities between ergot alkaloids and monoamine neurotransmitters (e.g., serotonin, norepinephrine, and dopamine), which elicit agonistic effects on numerous monoamine receptors [11,12,13,14,15]. While many livestock species do experience ergot alkaloid-induced effects on productivity, there is a high variability between individual animal responses to exposure. However, across livestock species, there have been consistent reports indicating that ergot alkaloids cause issues with reproductive performance, including reduced pregnancy rates, circulating hormone concentrations, blood flow to reproductive organs, and offspring birth weight [16]. Therefore, this review will focus on the various effects of ergot alkaloids (e.g., physiologic, mechanistic, etc.) on female reproduction in livestock species.

## 2. Ergot Alkaloids and the Brain

Ergot alkaloids share many structural similarities to monoamine neurotransmitters, and thus interact on the various monoamine receptors within the brain. Specifically, the cells within the anterior pituitary contain monoamine receptors and therefore ergot alkaloids may interfere with the physiological processes regulated by the anterior pituitary. The anterior pituitary can be divided into five endocrine cell types based on morphology and functional role: Somatotrophs (growth hormone (GH)), corticotrophs (adrenocorticotropic hormone (ACTH)), thyrotrophs (thyroid-stimulating hormone (TSH)), lactotrophs (prolactin (PRL)), and gonadotrophs (luteinizing hormone (LH) and follicle stimulating hormone (FSH)) [17]. While it does not appear that ergot alkaloids affect secretion of GH and TSH, there is great evidence that ergot alkaloids alter secretion of ACTH, PRL, and LH and FSH [12,18,19].

### 2.1. Dopamine Receptor D2 and Prolactin Secretion

A common symptom of fescue toxicosis is a suppression in prolactin secretion (i.e., hypoprolactinemia; [20,21]). Secretion of prolactin is predominantly regulated by hypothalamic prolactin-inhibiting factors, specifically the monoamine neurotransmitter dopamine [22]. Dopamine is considered the major regulator of prolactin secretion, and secretion is inhibited when dopamine is bound to its receptor on the lactotrophs of the anterior pituitary [23,24]. There are five isoforms of dopamine receptors that are divided into two subfamilies: D1-like, which is comprised of D1 and D5, and D2-like, which is comprised of D2, D3, and D4 [25]. On the pituitary lactotrophs, the dopamine receptors are primarily the dopamine receptor D2 (DRD2) [26]. The dopamine receptor D2 is coupled to a Giα protein, which inhibits adenylyl cyclase activity, cyclic adenosine monophosphate (cAMP), and cytoplasmic calcium concentrations when dopamine or an agonist is bound [27,28], thus resulting in a suppression of prolactin secretion.

Prolactin concentrations have been shown to be reduced in animals consuming endophyte-infected tall fescue and this is often used as an indicator of fescue toxicosis [10]. Ergot alkaloids, specifically ergovaline, have a high affinity towards DRD2 in vitro and elicit agonistic effects that result in a decrease in prolactin secretion [29]. Moreover, a recent study conducted gene expression profiles of pituitaries from steers grazing either high (HE; 746 parts per billion (ug/kg)) or low (LE; 23 ug/kg) endophyte-infected tall fescue pastures [19]. Li et al. [19] demonstrated DRD2 and PRL expression decreased in HE steers by 53% and 82%, respectively. Since PRL synthesis is directly related to DRD2 signaling pathway, a single nucleotide polymorphism (SNP) in the DRD2 gene could serve as a marker for resistance to fescue toxicosis as proposed by Campbell et al. [30].

Prolactin plays a role in numerous biological functions, most notably in lactation, but also in reproduction, immune responses, and metabolism [24]. Additionally, an elevation in prolactin is associated with a rise in environmental temperature or longer day length (i.e., photoperiod; [31,32]), and has been related to hair coat shedding in numerous species [33,34,35,36]. It has been speculated that hyperprolactinemia due to consumption of ergot alkaloid prevents shedding of the winter hair coat, therefore resulting in an elevation in core body temperature and increased vulnerability to heat stress (i.e., hyperthermia; [36]). In regards to reproduction, the heat stress-like signs greatly associated with fescue toxicosis have a large impact on reproductive success in livestock species and will be described in further detail in further sections of this review. Additionally, the interaction between ergot alkaloids and lactation, in which prolactin plays a critical role, will be discussed in greater detail in subsequent sections of this review.

### 2.2. Gonadotropins

The gonadotropins, luteinizing hormone (LH) and follicle stimulating hormone (FSH), are produced by the gonadotrophs of the anterior pituitary in response to gonadotropin releasing hormone (GnRH) secreted from the hypothalamus and govern reproductive cyclicity [37]. Browning Jr. et al. [38] demonstrated in steers receiving injections of ergotamine tartrate (23.8 µg/kg body weight) that LH concentrations were reduced. Therefore, it was then investigated if acute ergot alkaloid exposure (19 µg/kg body weight) would alter LH and FSH in primiparous cows during the late luteal phase (day 15 or 16 post-estrus). Luteinizing hormone concentrations were reduced 4 h post injection, however FSH concentrations did not differ between the cows receiving ergotamine tartrate and the saline control [39]. An in vitro study using ovine pituitary cells demonstrated that bromocriptine (ergocryptine derivative) inhibits LH and FSH secretion in response to GnRH [40]. Conversely, a separate study found no differences in LH secretion in postpartum beef cows nor in cycling heifers and cows [41]. Additional studies further described that ergot alkaloids do not suppress LH or equine chorionic gonadotropin (eCG) in ewes or mares, respectively [42,43]. Most recently, Li et al. [19] found minimal differences in pathways utilized for FSH and LH production, secretion, or signaling from pituitary tissue collected from steers grazing either HE or LE fescue pastures. Variations in ergot alkaloid source and concentration, route of administration, environmental conditions, and physiological status of the animal could account for these discrepancies, and thus additional research is needed to better understand the actions ergot alkaloids have on gonadotropin synthesis, secretion, and functionality.

## 3. Ergot Alkaloids and Ovarian Function

Reproductive failure in cattle following ergot alkaloid exposure can be attributed to altered ovarian follicle development, luteal dysfunction, and reduced circulating steroid hormone concentrations, subsequently leading to reduced pregnancy rates [44]. Conversely, ergot alkaloid exposure has minimal impact during reproductive cyclicity and a greater impact during pregnancy and post-partum in sheep and mares. Specifically, in the mare resulting in prolonged gestation, late-term foal loss, dystocia, thickened placentas, and agalactia [45], thus discussion on the effects of ergot alkaloids on ovarian function will emphasize the data collected on cattle.

### 3.1. Ovarian Blood Flow

Ergot alkaloids induce a vasoconstrictive response by interacting with biogenic amine receptors including serotonergic and adrenergic receptors [46]. Numerous studies have demonstrated ergot alkaloid, specifically ergovaline and ergotamine, induced vasoconstriction via the serotonin 2A (5-HT2A) receptor utilizing an in vitro bovine lateral saphenous vein bioassay [13,14]. Similarly, ergot alkaloids have a high affinity towards α_2_-adrenergic receptors [47,48]. Additionally, a couple of studies have used Doppler ultrasonography to show that heifers chronically exposed to endophyte-infected tall fescue have reduced caudal artery area and blood flow to the peripheral arteries when compared to heifers consuming endophyte-free tall fescue [49,50], however literature describing the extent to which vasoconstriction occurs to the internal organs is limited [16].

Recently, Poole et al. [50] investigated if chronic exposure of ergot alkaloids would decrease the diameter of the utero-ovarian blood vessels thus reducing systemic blood flow to the ovary during various stages of the estrous cycle. Ovarian artery and vein area was measured via Doppler ultrasonography on days 0, 4, 10, and 17 to represent both the follicular and luteal phases of the estrous cycle. Ovarian artery area was not different on days 0 and 4, however ovarian artery area was reduced on days 10 and 17 in heifers consuming ergot alkaloids. Additionally, minimal changes were observed in the ovarian vein area, most likely due to the reduction of vascular smooth muscle cells surrounding veins compared to arteries. Previous studies have demonstrated estrogen-induced vasodilation in various arteries and veins [51], and increased estrogen concentration early in the estrous cycle may have prevented the ergot alkaloid induced reduction in ovarian artery area that was observed during the luteal phase [50]. Ultimately, ergot alkaloid induced vasoconstriction of the utero-ovarian vessels would limit nutrients essential to ovarian function potentially altering sex steroid synthesis, altered follicular, and/or luteal development as well as dysregulation of the estrous cycle.

### 3.2. Folliculogenesis

As previously mentioned, the gonadotropins (LH and FSH) are produced by the anterior pituitary in response to GnRH and govern reproductive cyclicity [37]. Follicle stimulating hormone initiates the proliferation of granulosa cells and aid in the transition from primordial to primary and secondary follicles. As these follicles grow and undergo selection, granulosa cells become more responsive to LH and theca cells will produce androgens, which are converted primarily to estradiol (E2) in the granulosa cells [52,53]. As E2 and inhibin concentrations produced by the growing selected follicles increase, FSH production by the anterior pituitary is suppressed via negative feedback [54,55]. A few selected follicles will continue to grow into dominance and produce more E2, while others will become atretic. During the final follicular wave (post-luteolysis), large concentrations of E2 produced by the preovulatory follicle will act via positive feedback to trigger the preoptic nucleus of the hypothalamus to release a surge of GnRH, which will stimulate the anterior pituitary to release the preovulatory surge of LH and ovulation will occur [56,57,58].

McKenzie and Erickson [59,60] observed a decrease in the diameter and number of large follicles in heifers consuming endophyte-infected tall fescue (Table 1). Likewise, Burke and Rorie [61] examined follicular development and estrogen concentrations in lactating beef cows grazing endophyte-free (EF) or endophyte-infected (EI) tall fescue. No differences were found in the number of class 1 (small; 3 to 5 mm) and class 3 (large; >10 mm) antral follicles between treatments. Conversely, the number of class 2 (medium; 6–9 mm) follicles was reduced in cows grazing EI fescue compared to cows grazing EF fescue (Table 1). Similarly, Poole et al. [50] observed that a 6 to 9 mm follicle number was reduced in heifers consuming ergot alkaloids (Table 1). The 6 to 9 mm follicle size can be classified as selected follicles, and are of critical importance to follicular development with the gonadotropin dependence switching from FSH to LH. These results suggest that exposure to ergot alkaloids may hinder follicular selection though inadequate delivery of gonadotropins and other nutrients due to insufficient blood flow to the ovary.

Furthermore, many of these signs in cattle exposed to ergot alkaloids are amplified during periods of heat stress. The inability to maintain a thermoneutral body temperature due to increased ambient temperatures has been previously shown to impair folliculogenesis [65,66]. Therefore, Burke et al. [62] investigated the interaction between heat stress and consumption of endophyte-infected fescue on follicular dynamics. These authors controlled dietary intake for the heat stressed heifers on both diets to minimize variation in intake. Heifers consuming the EI seed in heat stress conditions resulted in a decreased number of large follicles (>9 mm) in addition to having a smaller preovulatory follicle diameter compared to control heifers (Table 1). Recently, a genetic trait has been identified in Bos taurus-influenced breeds, Senepol, and other Criollo cattle breeds, that is associated with high heat tolerance and a slick hair coat [67,68,69,70]. This slick hair coat phenotype is due to a frame-shift mutation in the prolactin receptor [70]. Therefore, Poole et al. [63] evaluated the effect of the slick trait and exposure to ergot alkaloids on follicular dynamics in beef heifers. Dietary intake and average daily gain (ADG) remained constant between groups. Heifers consuming the EI fescue with a wild-type hair coat (lacking the slick hair mutation) had an increase in the number of preselected follicles (2 to 4 mm), however, no change in the number of selected follicles (5 to 8 mm), yet a decrease in the number of preovulatory follicles (>9 mm) compared to the other heifer groups (wild-type hair coat consuming EF fescue and heifers with a slick-type hair coat consuming EI or EF fescue; Table 1). Intriguingly, this lack of follicular transition indicates a dysregulation during follicular selection during folliculogenesis that was not observed in heifers possessing the slick hair trait and consuming the EI fescue. Together, both Burke et al. [62] and Poole et al. [63] demonstrated that heat stress altered fescue toxicosis and alters the efficiency of ovarian follicular selection and dominance.

### 3.3. Corpus Luteum

The corpus luteum (CL) is a transient endocrine organ that forms on the ovary following ovulation. When a follicle ovulates, the granulosa and theca cells undergo dramatic changes into luteal cells, a process known as luteinization. Progesterone (P4) is the primary hormone produced by the CL and has numerous functions including suppression of ovulation and maintenance of pregnancy. It has been demonstrated that LH, not prolactin, is the predominant luteotropic hormone responsible for maintenance of the CL and production of P4 in livestock species [71,72,73]. Specifically, the functionality of the CL (i.e., production of P4) is dependent on the degree of vascularization or angiogenesis [74,75].

Due to the vasoconstrictive effects of ergot alkaloids, many researchers speculated that chronic exposure would result in luteal dysfunction, thus reducing pregnancy rates. Estienne et al. [76] observed a reduction in circulating P4 concentrations in heifers on endophyte-infected fescue even though no differences in CL size or presence were observed via ultrasonography. Interestingly, a separate study found that even if heifers appeared to be cycling and ovulating normally, there were cellular changes (fewer nuclei and a greater number of large luteal cells with increased diameter) of the CL in heifers grazing endophyte-infected fescue, which may contribute to altered functionality [77]. As previously mentioned, Poole et al. [63] observed a decrease in the diameter of the ovulatory follicle in EI heifers, and because of the process of luteinization it is not surprising that the luteal area (mm^2^) was also reduced in heifers consuming the EI fescue.

There have been varying reports regarding the impact of fescue toxicosis on luteal formation and function with numerous findings observing no differences [50,78,79]. This variation in responses could be due to the fact that circulatory ovarian steroid concentrations, specifically progesterone, are not only dependent on the rate of secretion but also on the metabolism in the liver and on the incidence of vasodilation or vasoconstriction.

### 3.4. Ovarian Steroidogenesis

One theory to explain the altered follicular dynamics and luteal dysfunction in animals consuming endophyte-infected fescue is a reduction in the steroid hormone (E2 and P4) precursor, cholesterol. Following synthesis in the liver from low-density and high-density lipoproteins; cholesterol must be transported to the ovary for sex steroid synthesis in the thecal, granulosal, and luteal cells [80,81]. Few studies have shown a decrease in circulating cholesterol concentrations in cattle consuming EI tall fescue [62,82,83]. Moreover, Burke et al. [62] observed that heat stress conditions further reduced cholesterol concentrations in heifers consuming EI fescue.

#### 3.4.1. Estradiol

Estrogen production is a critical component of a healthy developing follicle [52] and essential for reproductive success. Burke et al. [62] observed that E2 concentrations were reduced in heifers consuming EI fescue compared to the control heifers in the thermoneutral environment, whereas the additive effect of heat stress reduced E2 concentrations regardless of ergot alkaloid exposure. However, these results were not observed in postpartum cows consuming either EF or EI tall fescue [61]. Interestingly, Poole et al. [63] demonstrated that EI heifers with a wild-type hair coat (heat stressed) had decreased E2 concentrations, yet EI heifers possessing the slick hair trait had similar E2 concentrations to EF heifers. Collectively, ergot alkaloids impair follicular development and E2 secretion; however, it remains unknown if ergot alkaloids directly impact granulosal and thecal cell function or indirectly alter folliculogenesis through reduced blood flow to the ovary.

#### 3.4.2. Progesterone

Similar to the varying reports regarding the impact of fescue toxicosis on CL formation, there are contrasting reports regarding P4 synthesis and secretion from the CL. Mahmood et al. [84] examined luteal function in heifers grazing either low (0%) or high (>75%) EI fescue pastures for 168 days. Heifers were synchronized with prostaglandin F_2α_ (PGF_2α_) on days 101 and 112 of the trial and P4 concentrations were determined on days 112, 116, 120, and 124. Heifers on the high EI fescue pastures had either low P4 concentrations (<1.5 ng/mL) after synchronization or relatively high P4 concentrations (>1.5 ng/mL) that would sharply decrease, thus indicating luteal dysfunction or shorten luteal phase [85], respectively. Both Burke et al. [62] and Poole et al. [63] demonstrated that heat stress exacerbated fescue toxicosis and observed a reduction in P4 concentrations. Conversely, numerous reports have found no differences in P4 secretion in animals consuming EI fescue [50,78,79]. Interestingly, Jones et al. [86] evaluated P4 concentrations in EF, EI, and EI heifers treated with domperidone (EID; dopamine antagonist) during the months of May and June in Southern Illinois. Heifers consuming EI fescue had reduced mid-cycle P4 concentrations when compared to EF and EID heifers. Furthermore, cultured luteal cells collected from a subset of heifers from each treatment group revealed no differences observed in P4 secretion in vitro [86]. The authors suggest that utilization of domperidone in vivo may alleviate some of the signs associated with fescue toxicosis (i.e., reduced PRL and heat stress) to improve CL function.

## 4. Ergot Alkaloids and Uterine Function

### 4.1. Uterine Blood Flow

Dyer [87] was the first to report the interaction between ergovaline and serotonin (5-HT) receptors in bovine uterine arteries. Furthermore, Poole et al. [50] measured uterine artery and vein area via Doppler ultrasonography on days 0, 4, 10, and 17 of the estrous cycle in EF and EI beef heifers. It was observed that uterine artery and vein areas were not different on days 0 and 4, however, uterine artery and vein areas did differ on days 10 and 17 with heifers consuming ergot alkaloids and this is the time of the estrous cycle when P4 concentrations are greatest. The ergot alkaloids induced vasoconstriction of the uterine vessels occurred prior to the timing of maternal recognition of pregnancy (day 14–16) in cattle, Poole et al. [50] speculated that this could reduce hormonal communication between the ovary and uterus during this time of embryonic signaling to the endometrium, thus decrease pregnancy retention [88]. Vasoconstrictive activity has also been detected in ovine uterine arteries [89]. Pregnant Suffolk ewes were subjected to either an EF or EI (1770 µg/day ergovaline) diet from day 35 to 86 of gestation (Period 1), then were fed the same diet either throughout or received a crossover diet with fescue type opposite of the original diet until termination of pregnancy on day 133 of gestation (Period 2). Ewes fed EI fescue during Period 2 had reduced uterine vessel area, however ewes fed EI fescue during Period 1 then switched to EF fescue did not display this reduced vessel area. Moreover, utilizing an in vitro bioassay the uterine arteries collected from EF ewes during Period 2 were responsive to serotonin and ergot alkaloids (ergotamine and ergovaline) [89]. In regards to the mare, uterine arteries collected from non-pregnant mares were subjected to an in vitro bioassay, however, were not responsive to serotonin, ergotamine, or ergovaline [90]. Klotz and McDowell [90] suggested that effects of ergot alkaloids on reproductive failure in the mare might not be a consequence of vasoconstriction and restricted blood flow.

### 4.2. Prostaglandin F_2α_ (PGF_2α_) Synthesis

It has been suggested that ergot alkaloids have an oxytocic effect (i.e., contractile response) on the uterus [91]. During late luteal phase of the estrous cycle, oxytocin acts on localized receptors on the endometrium to stimulate synthesis of PGF_2α_, thus triggering luteolysis [92,93]. A few studies have evaluated the effect of ergot alkaloids on PGF_2α_ secretion. Browning Jr. et al. [39] injected ergotamine tartrate to primiparous cows during the late luteal phase (day 15 or 16 post-estrus) and observed that PGF_2α_ concentrations were elevated just one hour post injection and continued to increase every hour for four hours. This response mirrored that of pulsatile PGF_2α_ response observed during luteolysis; unfortunately, the authors did not evaluate luteal function or regression. Vogt Engeland et al. [94] administered 105 µg ergotamine per kilogram of body weight via oral drench twice daily from day 98 to 107 of gestation in dairy goats, and observed that ergotamine treated goats had significantly greater concentrations of PGF_2α_ resulting in a greater incidence of induced parturition and fetal death [94].

## 5. Ergot Alkaloids and Pregnancy

**Figure 1:**
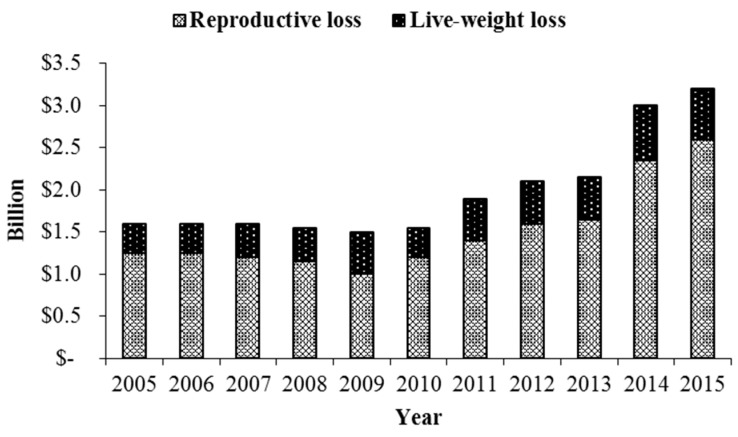
Fescue toxicosis severely reduces calving rates with 16% of reproductive beef cows failing to conceive or experiencing early embryonic loss. Calves born in a fescue environment have an average reduction in weaning weight by 22.6 kg (Reproduced from Kallenbach [9]. 2015, Oxford University Press).

A common symptom of fescue toxicosis is reduced pregnancy rates, specifically in cattle [9,10,95]). According to a review by Kallenbach [9], fescue toxicosis attributes to over $2 billion in annual economic loss to the U.S. livestock industries, primarily due to reproductive loss (Figure 1). In cattle, this reproductive loss is because of a failure to conceive or early embryonic loss [9]. Additionally, many livestock species experience difficulties during late-gestation and this has an impact on fetal and neonatal development [9,45,96].

### 5.1. Early Embryonic Development

A few studies have evaluated the impact that ergot alkaloids have on oocyte competency and early embryonic development. Jones et al. [97] cultured cumulus-oocyte-complexes (COCs) with either a control medium, EF-treated medium with 10% EF plasma, or EI-treated medium with 10% EI plasma supplemented. Plasma used as a treatment was previously collected when the heifers were exposed to EF or EI pastures for 24 days. There were no differences in the percent of COCs that progressed to metaphase II (MII). Additionally, ovum pick-up was performed on the heifers and the grade I oocytes (≥5 layers of compact cumulus cells and homogenous cytoplasm; [98]) were subjected to traditional in vitro procedures. Interestingly, there was a difference observed with 66% of the EF grade I oocytes progressing to MII versus 0% of the EI grade I oocytes, thus demonstrating that in vivo exposure to endophyte-infected tall fescue can directly inhibit proper oocyte maturation [97].

Schuenemann et al. [99] explored the impact of ergot alkaloids on early embryonic development (experiment 1) and uterine receptivity (experiment 2) in vivo. Cattle were allotted to receive either the control (CON) or an ergot alkaloid seed (EI) diet. In experiment 1, uterine horn ipsilateral to the CL was flushed for embryo recovery following estrous synchronization and artificial insemination. Embryo recovery tended to be more successful in CON cattle versus EI cattle. Of the embryos recovered, a greater percent of embryos from CON animals had developed to compacted morula or blastocyst, and there was a greater percent of better quality embryos from CON cattle versus ET cattle [99]. In experiment 2, two frozen-thawed good quality embryos were transferred to recipients in both treatment groups seven days following synchronized estrus. Interestingly, pregnancy rates following transfer did not differ [99]. The authors concluded that the uterine environment is suitable to maintain pregnancy after day 7 of gestation, however ergot alkaloid exposure appears to detrimentally affect either the oocyte or the early embryo prior to the blastocyst stage.

As previously mentioned, elevated body temperature and heat stress-like signs are greatly associated with cattle exposed to ergot alkaloids. It is also well established that heat stress can have a negative effect on most aspects of female reproduction including oogenesis, oocyte maturation, and early embryonic development [100]. In fact, results from numerous studies evaluating heat stress and early embryonic development mirror the results observed by Schuenemann et al. [99]. For example, Ealy et al. [101] observed that exposure to heat stress conditions at day 1 post-estrus (two-cell cleaved embryos), reduced the percent of embryos that developed to the blastocyst stage. However, heat stress exposure at days 3, 5, and 7 had no effect on the percent of embryos that were blastocysts. Likewise, Edwards and Hansen [102] found that heat stress did not impact oocytes during the first 12 h of maturation, however it greatly reduced the number of two-cell embryos that developed to the blastocyst stage in vitro. While ergot alkaloid exposure inhibits early embryonic development in vitro, in combination with the inability to maintain a thermoneutral body temperature due to heat stress severely impacts embryonic development resulting in decreased pregnancy rates in many spring calving herds throughout the Southeastern and Mid-Atlantic states.

Interestingly, a few studies have evaluated effects on embryonic development at levels greater than 300 μg/kg in mares (Table 2). At 867 μg/kg ergovaline, there was no impact on embryonic development or establishment of pregnancy in mares [43]. However, at 1171 μg/kg, there was an increased incidence of early embryonic loss and reduced pregnancy rates in mares [103]. Minimal effects were observed if mares were exposed to less than 300 μg/kg ergovaline [104]. Unlike cattle exposed to ergot alkaloids, mares do not experience elevated body temperatures, which is most likely due to evaporative cooling because of increased sweating capability [105], which may mitigate the negative effects of ergot alkaloids on early embryo loss. The effects of ergot alkaloids on pregnant mares are much greater during late gestation [106], suggesting that ergot alkaloids potentially impact placental efficiency.

### 5.2. Placenta

It has been shown that ergot alkaloids can cross the placental barrier in rodents [109,110], but conclusive evidence in livestock species is unavailable. However, similar to the uterine artery, there have been reports that ergot alkaloids induce a vasoconstrictive response in both bovine and ovine umbilical arteries [87,89]. Britt et al. [111] examined placental characteristics in pregnant Suffolk ewes following exposure to EF or EI (1770 µg/day ergovaline) diets and found that ewes subjected to an EI diet during Period 2 experienced an overall reduction in total caruncle, cotyledon, and placentome weight. In sheep, the placenta increases in vascularity after day 80 of gestation to support rapid fetal growth [112,113]. Therefore, it is believed that this vasoconstrictive activity on the umbilical artery reduces blood flow to the placenta and subsequently the fetus, which results in reduced birth weights [89,111]. Ergot alkaloid induced effects on the placenta are evident in the mare. Early reports suggested that pregnant mares grazing endophyte-infected tall fescue experience reproductive failure [114,115]. Specifically, Monroe et al. [116] demonstrated that mares grazing EI tall fescue during late gestation experienced prolonged gestation, increased number of retained placentas, and increased placental weight and thickness when compared to mares grazing EF tall fescue. Due to these findings, it is now highly recommended that pregnant mares are removed from EI tall fescue pastures during the third trimester to avoid any serious complications.

### 5.3. Fetal Programming

Studies have found that ergot alkaloid exposure during gestation results in a reduction in birth weight in lambs [96,111] and calves [117,118]. There are a few theories to explain this reduction in birth weight. One being the previously described vasoconstriction to the uterine and placental arteries, however another is a decrease in daily nutrient intake by the dam, thus resulting in a reduction in dam body weight [111,118]. Either mechanism results in a reduction in placental growth and supply of nutrients to the developing fetus. Placental growth is partly regulated by the paternally imprinted gene, insulin-like growth factor-2 (IGF2), and when there are modifications or changes in expression of imprinted genes, then this is associated with developmental programming and a reduction in birth weight [119,120]. Interestingly, Britt et al. [111] found that ewes exposed to ergot alkaloids during Period 1 and 2 of gestation had increased mRNA expression of IGF2 in cotyledon tissue, however these differences were not observed in ewes only exposed to ergot alkaloids during Period 1 or 2. The authors speculated that these adverse conditions (i.e., exposure to ergot alkaloids) resulted in the increase in IGF2 expression to aid in placental adaptation to the conditions [111].

## 6. Lactation

The effects of ergot alkaloids on lactation vary based on the livestock species. Consumption of ergot alkaloids reduces milk yield in cattle and sheep [44]. However, while prolactin plays a critical role in mammary gland development and milk synthesis [121], it is important to note that decreased prolactin concentrations does not directly influence milk yield in these species [122] and ergot alkaloid exposure during the dry period does not impair mammary development or milk production in the following lactation [123]. More recently, Capuco et al. [124] described changes in the mammary gland transcriptome related to lipid metabolism, and molecular transport following exposure to endophyte-infected fescue seed, which could contribute to reduced milk production as previously reported by Baldwin et al. [123]. However, cofounding factors such as reduced feed intake following exposure to endophyte-infected fescue seed in these studies [123,124] limits identification of the exact mechanism of action of ergot alkaloids on the mammary gland. Therefore, other associated signs of fescue toxicosis, such as reduced feed intake or vasoconstriction, play a more critical role in the reduction of milk production in cattle and sheep. In contrast, horses exhibit complete agalactia when exposed to ergot alkaloids [45]. Unlike ruminants, which produce both placental lactogen and prolactin to initiate prepartum lactogenesis, horses rely solely on prolactin [125]. Thus, horses become agalactic when grazing endophyte-infected tall fescue due to ergot alkaloids agonistic effects of DRD2 and subsequent decrease in prolactin secretion. Many proposed strategies are available to mitigate the signs of fescue toxicosis in mares, but perhaps the one of the most effective to improve lactation is administering a dopamine antagonist, domperidone [45]. Redmond et al. [126] conducted a study to determine the minimum effective oral dose of domperidone (1.1, 1.65, 2.2 mg/kg BW) to treat fescue toxicosis in late-gestation mares. Redmond et al. [126] concluded that the minimum oral dose of 1.1 mg/kg BW was effective to alleviate signs of fescue toxicosis when provided daily for 30 days before foaling. A follow-up study determined that subcutaneous administration of 0.44 mg/kg BW domperidone 10 days before foaling was also effective to alleviate signs and improve lactation in mares [127]. While domperidone is an effective alleviator of agalactia in mares grazing EI tall fescue, most veterinarians would recommend removal from EI tall fescue pastures at least 30 days before the expected foaling date [45].

## 7. Conclusions

While evidence of reduced reproductive performance of animals consuming endophyte-infected tall fescue has been extensively studied in an attempt to find remedies for, or offset the negative impact of, fescue toxicosis; the complex etiology of this syndrome has hindered an exploration of specific mechanisms of action of ergovaline on specific tissues. Seasonal or annual fluctuations in ergot alkaloid concentrations in combination with the age and genetic background of the animal, elevated environmental conditions, and/or hypoxic conditions at the cellular level influence the impact ergot alkaloids have on the reproductive tissues leading to inconsistencies in reduced reproductive performance in animals consuming ergot alkaloid-contaminated diets. Further exploration into the precise mechanism of action of ergot alkaloids on the hypothalamic-pituitary-gonadal axis through innovative research combining cellular and molecular techniques with applied experimental models will lead to a better understanding of the negative impact these toxins on reproductive processes. Moreover, this knowledge will lead to inventive tools and strategies enhance best management practices to improve reproductive performance in animals consuming endophyte-infected tall fescue.

## Figures and Tables

**Table 1:** A review of the effects of ergot alkaloids and/or heat stress on follicular dynamics.

Alkaloid (µg/kg/day)	Heat Stress	Average Daily Gain	Animal	Effect	Source
N/A	N/A	N/A	Beef heifer	Decrease in the diameter and number of large follicles.	[59]
N/A	N/A	N/A	Beef heifer	Decrease in the diameter and number of large follicles.	[60]
N/A	Possibly, grazing from April to Sept. in Arkansas	Reduced	Mature beef cows	Diameter of the largest follicle tended to be smaller. No difference in the number of small or large follicles. Number of medium sized follicles reduced.	[61]
10,310 total; 3910 ergovaline	Possibly, barn temperature. 27 °C and average THI ^1^ 72	Reduced, same intake	Beef heifer	Number of medium sized follicles reduced. No difference in the number of small or large follicles or diameter of largest follicle.	[50]
1900 ergovaline	Yes, between 25 °C and 31 °C	N/A, reduced intake	Beef heifer	Decrease in the diameter and number of large follicles.	[62]
1000 total; 421 ergovaline	Yes, average THI 88	Same	Beef heifer	Decrease in the diameter and number of small and large follicles.	[63]

^1^ THI = temperature-humidity index; THI = (1.8 × T_db_ +32) – ((0.55 – 0.0055 × RH) × (1.8 × T_db_ – 26.8)); T_db_ = dry bulb temperature (°C), and RH = relative humidity. Recovery (non-life threatening, 75) and emergency (high-risk, 85) thresholds as described by Hahn [64].

**Table 2:** A review of the effects of ergovaline on early embryonic development and establishment of pregnancy in the mare.

Ergovaline (μg/kg)	Effect	Source
45	No negative effects	[107]
160	No negative effects	[108]
271	No negative effects	[107]
308	Suppressed PRL, no negative effects on pregnancy outcomes	[108]
867	Decreased P4, no negative effects on pregnancy outcomes	[43]
1171	Increase in early embryonic loss and reduced pregnancy rates	[104]

## References

[1] Jakubczyk D., Cheng J.Z., O’Connor S.E. (2014). Biosynthesis of the ergot alkaloids. Nat. Prod. Rep..

[2] Bacon C., Porter J., Robbins J., Luttrell E. (1977). *Epichloë typhina* from toxic tall fescue grasses. Appl. Environ. Microbiol..

[3] Glenn A.E., Bacon C.W., Price R., Hanlin R.T. (1996). Molecular phylogeny of *Acremonium* and its taxonomic implications. Mycologia.

[4] Leuchtmann A., Bacon C.W., Schardi C.L., White J.F., Tadych M. (2014). Nomenclatural realignment of *Neotyphodium* species with genus *Epichloë*. Mycologia.

[5] Lyons P.C., Plattner R.D., Bacon C.W. (1986). Occurrence of peptide and clavinet ergot alkaloids in tall fescue grass. Science.

[6] Burns J.C., Chamblee D.S., Buckner R., Bush L. (1979). Adaptation. Tall Fescue. Agronomy Monographs 20.

[7] Strickland J.R., Aiken G.E., Spiers D.E., Fletcher L.R., Oliver J.W., Fribourg H.A., Hannaway D.B., West C.P. (2009). Physiological Basis of Fescue Toxicosis. Tall Fescue for the Twenty-First Century. Agronomy Monographs 53.

[8] Siegel M.R., Latch G.C.M., Johnson M.S. (1985). *Acremonium* fungal endophytes of tall fescue and perennial ryegrass: Significance and control. Plant Dis..

[9] Kallenbach R.L. (2015). BILL E. KUNKLE INTERDISCIPLINARY BEEF SYMPOSIUM: Coping with tall fescue toxicosis: Solutions and realities. J. Anim. Sci..

[10] Strickland J.R., Looper M.L., Matthews J.C., Rosenkrans C.F., Flythe M.D., Brown K.R. (2011). BOARD-INVITED REVIEW: St. Anthony’s fire in livestock: Causes, mechanisms, and potential solutions. J. Anim. Sci..

[11] Berde B., Goldstein M., Lieberman A., Calne D.B., Thorner M.O. (1980). Ergot compounds: A synopsis. Ergot Compounds and Brain Function: Neuroendocrine and Neuropsychiatric Aspects.

[12] Elsasser T.H., Bolt D.J. (1987). Dopaminergic-like activity in toxic fescue alters prolactin but not growth hormone or thyroid stimulating hormone in ewes. Domest. Anim. Endocrinol..

[13] Klotz J.L., Brown K.R., Xue Y., Matthews J.C., Boling J.A., Burris W.R., Bush L.P., Strickland J.R. (2012). Alterations in serotonin receptor-induced contractility of bovine lateral saphenous vein in cattle grazing endophyte-infected tall fescue. J. Anim. Sci..

[14] Klotz J.L., Aiken G.E., Johnson J.E., Brown K.R., Bush L.P., Strickland J.R. (2013). Antagonism of lateral saphenous vein serotonin receptors from steers grazing endophyte-free, wild-type, or novel endophyte-infected tall fescue. J. Anim. Sci..

[15] Klotz J.L., Aiken G.E., Bussard J.R., Foote A.P., Harmon D.L., Goff B.M., Schrick F.N., Strickland J.R. (2016). Vasoactivity and Vasoconstriction Changes in Cattle Related to Time off Toxic Endophyte-Infected Tall Fescue. Toxins.

[16] Klotz J.L. (2015). Activities and Effects of Ergot Alkaloids on Livestock Physiology and Production. Toxins.

[17] Nakane P.K. (1970). Classifications of anterior pituitary cell types with immunoenzyme histochemistry. J. Histochem. Cytochem..

[18] Thompson F.N., Stuedemann J.A., Sartin J.L., Belesky D.P., Devine O.J. (1987). Selected hormonal changes with summer fescue toxicosis. J. Anim. Sci..

[19] Li Q., Hegge R., Bridges P.J., Matthews J.C. (2017). Pituitary genomic expression profiles of steers are altered by grazing of high vs. low endophyte-infected tall fescue forages. PLoS ONE.

[20] Nasr H., Pearson O.H. (1975). Inhibition of prolactin secretion by ergot alkaloids. Acta Endocrinol..

[21] Hurley W.L., Convey E.M., Leung K., Edgerton L.A., Hemken R.W. (1980). Bovine prolactin, TSH, T and T concentrations as affected by tall fescue summer toxicosis and temperature. J. Anim. Sci..

[22] Ben-Johnathan N. (1985). Dopamine: A prolactin-inhibiting hormone. Endocr. Rev..

[23] Leong D.A., Frawley L.S., Neill J.D. (1983). Neuroendocrine control of prolactin secretion. Annu. Rev. Physiol..

[24] Freeman M.E., Kanyicska B., Lerant A., Nagy G. (2000). Prolactin: Structure, Function, and Regulation of Secretion. Physiol. Rev..

[25] Sibley D.R., Monsma F.J. (1992). Molecular biology of dopamine receptors. Trends Pharmacol. Sci..

[26] Caron M.C., Beaulieu M., Raymond V., Gange B., Drouin J., Lefkowitz J., Labrie F. (1978). Dopaminergic receptors in the anterior pituitary gland. J. Biol. Chem..

[27] Enjalbert A., Bockaert J. (1983). Pharmacological characterization of D2 dopamine receptors negatively coupled with adenylate cyclase in the rate anterior pituitary. Mol. Pharmacol..

[28] Lledo P.M., Homburger V., Bockaert J., Vincent J.D. (1992). Differential G Protein-Mediated Coupling of D2 Dopamine Receptors to K+ and Ca2+ Currents in Rat Anterior Pituitary Cells. Neuron.

[29] Larson B.T., Samford M.D., Camden J.M., Piper E.L., Kerley M.S., Paterson J.A., Turner J.T. (1995). Ergovaline binding and activation of D2 dopamine receptors in GH4ZR7 cells. J. Anim. Sci..

[30] Campbell B.T., Kojima C.J., Cooper T.A., Bastin B.C., Wojakiewicz L., Kallenbach R.L., Schrick F.N., Waller J.C. (2014). A single nucleotide polymorphism in the dopamine receptor D2 gene may be informative for resistance to fescue toxicosis in angus-based cattle. Anim. Biotechnol..

[31] Schams D., Reinhardt V. (1974). Influence of the season on plasma prolactin levels in cattle from birth to maturity. Hormone Res..

[32] Wettemann R.P., Tucker H.A. (1974). Relationship of ambient temperature to serum prolactin in heifers. Proc. Soc. Exp. Biol. Med..

[33] Thompson D.L., Hoffman R., DePew C.L. (1997). Prolactin administration to seasonally anestrous mares: Reproductive, metabolic, and hair-shedding responses. J. Anim. Sci..

[34] Foitzik K., Krause K., Nixon A.J., Ford C.A., Ohnemus U., Pearson A.J., Paus R. (2003). Prolactin and its receptor are expressed in murine hair follicle epithelium show hair cycle-dependent expression, and induce catagen. Am. J. Pathol..

[35] Nixon A.J., Ford C.A., Wildermoth J.E., Craven A.J., Ashby M.G., Pearson A.J. (2002). Regulation of prolactin receptor expression in ovine skin in relation to circulating prolactin and wool follicle growth status. J. Endocrinol..

[36] Aiken G.E., Klotz J.L., Looper M.L., Tabler S.F., Schrick F.N. (2011). Disrupted hair follicle activity in cattle grazing endophyte-infected tall fescue in the summer insulates core body temperatures. Prof. Anim. Sci..

[37] Hansel W., Convey E.M. (1983). Physiology of the Estrous Cycle. J. Anim. Sci..

[38] Browning R., Thompson F.N., Sartin J.L., Leite-Browning M.L. (1997). Plasma concentrations of prolactin, growth hormone, and luteinizing hormone in steers administered ergotamine or ergonovine. J. Anim. Sci..

[39] Browning R., Schrick F.N., Thompson F.N., Wakefield T. (1998). Reproductive hormonal repsonses to ergotamine and ergonovine in cows during the luteal phase of the estrous cycle. J. Anim. Sci..

[40] Hodson D.J., Henderson H.L., Townsend J., Tortonese D.J. (2012). Photoperiodic modulation of the suppressive actions of prolactin and dopamine on the pituitary gonadotropin responses to gonadotropin-releasing hormone in sheep. Biol. Reprod..

[41] Mizinga K.M., Thompson F.N., Stuedemann J.A., Kiser T.E. (1992). Effects of feeding diets containing endophyte-infected fescue seed on luteinizing hormone secretion in postpartum beef cows and in cyclic heifers and cows. J. Anim. Sci..

[42] Louw B.P., Lishman A.W., Botha W.A., Baumgartner J.P. (1974). Failure to demonstrate a role for the acute release of prolactin at oestrus in the ewe. J. Reprod. Fertil..

[43] Brendemuehl J.P., Carson R.L., Wenzel J.G.W., Boosinger T.R., Shelby R.A. (1996). Effects of grazing endophyte-infected tall fescue on eCG and progestogen concentrations from gestation days 21 to 300 in the mare. Theriogenology.

[44] Porter J.K., Thompson F.N. (1992). Effect of Fescue Toxicosis on Reproduction in Livestock. J. Anim. Sci..

[45] Cross D.L., Redmond L.M., Strickland J.R. (1995). Equine Fescue Toxicosis: Signs and Solutions. J. Anim. Sci..

[46] Pertz H.H., Eich E., Kren V., Cvak L. (1999). Ergot alkaloids and their derivatives as ligands for serotoninergic, dopaminergic, and adrenergic receptors. Ergot.

[47] McPherson G.A., Beart P.M. (1983). The selectivity of some ergot derivatives for alpha 1 and alpha 2-adrenoceptors of rat cerebral cortex. Eur. J. Pharmacol..

[48] Oliver J.W., Strickland J.R., Waller J.C., Fribourg H.A., Linnabary R.D., Abney L.K. (1998). Endophytic fungal toxin effect on adrenergic receptors in lateral saphenous veins (cranial branch) of cattle grazing tall fescue. J. Anim. Sci..

[49] Aiken G.E., Kirch B.H., Strickland J.R., Bush L.P., Looper M.L., Schrick F.N. (2007). Hemodynamic responses of the caudal artery to toxic tall fescue in beef heifers. J. Anim. Sci..

[50] Poole D.H., Lyons S.E., Poole R.K., Poore M.H. (2018). Ergot alkaloids induce vasoconstriction of bovine uterine and ovarian blood vessels. J. Anim. Sci..

[51] Miller V.M., Duckles S.P. (2008). Vascular actions of estrogens: Functional implications. Pharmacol. Rev..

[52] Fortune J.E., Quirk S.M. (1988). Regulation of steroidogenesis in bovine preovulatory follicles. J. Anim. Sci..

[53] Richards J.S. (1994). Hormonal control of gene expression in the ovary. Endocr. Rev..

[54] Law A.S., Logue D.N., O’Shea T., Webb T. (1990). Evidence for a novel factor in steroid free bovine follicular fluid (bFF) which acts to directly suppress follicular development. J. Reprod. Fertil. Abstr. Ser..

[55] Turzillo A.M., Fortune J.E. (1990). Suppression of the secondary FSH surge with bovine follicular fluid is associated with delayed ovarian follicular development in heifers. J. Reprod. Fertil..

[56] Wettemann R.P., Hafs H.D., Edgerton L.A., Swanson L.V. (1972). Estradiol and progesterone in blood serum during the bovine estrous cycle. J. Anim. Sci..

[57] Kinder J.E., Garcia-Winder M., Imakawa K., Day M.L., Zalesky D.D., D’Occhio M.J., Kittok R.J., Schanbacher B.D. (1983). Influence of different estrogen doses on concentrations of serum LH in acute and chronic ovariectomized cows. J. Anim. Sci..

[58] Day M.L., Imakawa K., Garcia-Winder M., Kittok R.J., Schanbacher B.D., Kinder J.E. (1986). Influence of prepubertal ovariectomy and estradiol replacement therapy on secretion of luteinizing hormone before and after pubertal age in heifers. Dom. Anim. Endocrinol..

[59] McKenzie P.P., Erickson B.H. (1989). The effects of fungal-infected fescue on hormonal secretion and ovarian development in the beef heifer. J. Anim. Sci..

[60] McKenzie P.P., Erickson B.H. (1991). Effects of fungal-infested fescue on gonadotrophin secretion and folliculogenesis in beef heifers. J. Anim. Sci..

[61] Burke J.M., Rorie R.W. (2002). Changes in ovarian function in mature beef cows grazing endophyte infected tall fescue. Theriogenology.

[62] Burke J.M., Spiers D.E., Kojima F.N., Perry G.A., Salfen B.E., Wood S.L., Patterson D.J., Smith M.F., Lucy M.C., Jackson W.G. (2001). Interaction of endophyte-infected fescue and heat stress on ovarian function in the beef heifer. Biol. Reprod..

[63] Poole R.K., Devine T.L., Mayberry K.J., Eisemann J.H., Poore M.H., Long N.M., Poole D.H. (2019). Impact of slick hair trait on physiological and reproductive performance in beef heifers consuming ergot alkaloids from endophyte-infected tall fescue. J. Anim. Sci..

[64] Hahn G.L. (1999). Dynamic response of cattle to thermal heat loads. J. Anim. Sci..

[65] Murphy M.G., Enright J.W., Crowe M.A., McConnell K., Spicer L.J., Boland M.P., Roche J.F. (1991). Effect of dietary intake on pattern of growth of dominant follicles during the oestrous cycle in beef heifers. J. Reprod. Fertil..

[66] Roth Z., Meidan R., Shaham-Albalancy A., Braw-Tal R., Wolfenson D. (2001). Delayed effect of heat stress on steroid production in medium-sized and preovulatory bovine follicles. Reproduction.

[67] Olson T.A., Lucena C., Chase C.C., Hammond A.C. (2003). Evidence of a major gene influencing hair length and heat tolerance in *Bos taurus* cattle. J. Anim. Sci..

[68] Mariasegaram M., Chase C.C., Chaparro J.X., Olson T.A., Brenneman R.A., Niedz R.P. (2007). The slick hair coat locus maps to chromosome 20 in Senepol derived cattle. Anim. Genet..

[69] Porto-Neto L.R., Bickhart D.M., Landaeta-Hernandez A.J., Utsunomiya Y.T., Pagan M., Jimenez E., Hansen P.J., Dikmen S., Schroeder S.G., Kim E. (2018). Convergent Evolution of Slick Coat in Cattle through Truncation Mutations in the Prolactin Receptor. Front. Genet..

[70] Littlejohn M.D., Henty K.M., Tiplady K., Johnson T., Harland C., Lopdell T., Sherlock R.G., Li W., Lukefahr S.D., Shanks B.C. (2014). Functionally reciprocal mutations of the prolactin signaling pathway define hairy and slick cattle. Nat. Commun..

[71] Kaltenbach C.C., Graber J.W., Niswender G.D., Nalbandov A.V. (1968). Luteotrophic properties for some pituitary hormones in nonpregnant or pregnant hypophysectomized ewes. Endocrinology.

[72] Murdoch W.J., Dailey R.A., Inskeep E.K. (1981). Preovulatory changes in prostaglandins E2 and F2α in ovine follicles. J. Anim. Sci..

[73] Farin C.E., Moeller C.L., Mayan H., Gamboni F., Sawyer H.R., Niswender G.D. (1988). Effect of luteinizing hormone and human chorionic gonadotropin on cell populations in the ovine corpus luteum. Biol. Reprod..

[74] Reynolds L.P., Grazul-Bilska A.T., Redmer D.A. (2000). Angiogenesis in the corpus luteum. Endocrine.

[75] Stocco C., Telleria C., Gibori G. (2007). The Molecular Control of Corpus Luteum Formation, Function, and Regression. Endocr. Rev..

[76] Estienne M.J., Schillo K.K., Fitzgerald B.P., Hielman S.M., Bradley N.W., Boling J.A. (1991). Effects of endophyte-infected fescue on puberty onset and luteal activity in beef heifers. J. Anim. Sci..

[77] Ahmed N.M., Schmidt S.P., Arbona J.R., Marple D.N., Bransby D.I., Carson R.L., Coleman D.A., Rahe C.H. (1990). Corpus luteum function in heifers grazing endophyte-free and endophyte-infected Kentucky-31 tall fescue. J. Anim. Sci..

[78] Fanning M.D., Spitzer J.C., Cross D.L., Thompson F.N. (1992). A preliminary study of Growth, serum prolactin and reproductive performance of beef heifers grazing *Acremonium coenophialum*-infected tall fescue. Theriogenology.

[79] Seals R.C., Schuenemann G.M., Lemaster J.W., Saxton A.M., Waller J.C., Schrick F.N. (2005). Follicular dynamics in beef heifers consuming ergotamine tartrate as a model of endophyte-infected tall fescue consumption. J. Anim. Vet. Adv..

[80] Pate J.L., Condon W.A. (1982). Effects of serum and lipoproteins on steroidogenesis in cultured bovine luteal cells. Mol. Cell. Endocrinol..

[81] Pate J.L., Condon W.A. (1989). Regulation of steroidogenesis and cholesterol synthesis by prostaglandin F-2 alpha and lipoproteins in bovine luteal cells. J. Reprod. Fertil..

[82] Rice R.L., Blodgett D.J., Schurig G.G., Swecker W.S., Fontenot J.P., Allen V.G., Akers R.M. (1997). Evaluation of humoral immune response in cattle grazing endophyte-infected or endophyte-free fescue. Vet. Immunol. Immunopathol..

[83] Nihsen M.E., Piper E.L., West C.P., Crawford R.J., Denard T.M., Johnson Z.B., Roberts C.A., Spiers D.A., Rosenkrans C.F. (2004). Growth rate and physiology of steers grazing tall fescue inoculated with novel endophytes. J. Anim. Sci..

[84] Mahmood T., Ott R.S., Foley G.L., Zinn G.M., Schaeffer D.J., Kesler D.J. (1994). Growth and ovarian function of weanling and yearling beef heifers grazing endophyte-infected tall fescue pastures. Theriogenology.

[85] Odde K.G., Ward H.S., Kiracofe G.H., McKee R.M., Kittok R.J. (1980). Short estrous cycles and associated serum progesterone levels in beef cows. Theriogenology.

[86] Jones K.L., King S.S., Griswold K.E., Cazac D., Cross D.L. (2003). Domperidone can ameliorate deleterious reproductive effects and reduced weight gain associated with fescue toxicosis in heifers. J. Anim. Sci..

[87] Dyer D.C. (1993). Evidence that ergovaline acts on serotonin receptors. Life Sci..

[88] Roberts R.M., Xie S., Mathialagan N. (1996). Maternal recognition of pregnancy. Biol. Reprod..

[89] Klotz J.L., Britt J.L., Miller M.F., Snider M.A., Aiken G.E., Long N.M., Pratt S.L., Andrae J.G., Duckett S.K. (2019). Ergot alkaloid exposure during gestation alters: II. Uterine and umbilical artery vasoactivity. J. Anim. Sci..

[90] Klotz J.L., McDowell K.J. (2017). Tall fescue ergot alkaloids are vasoactive in equine vasculature. J. Anim. Sci..

[91] Saameli K., Berde B., Schild H.O. (1978). Effects on the uterus. Ergot Alkaloids and Related Compounds. Handbook of Experimental Pharmacology.

[92] Wathers D.C., Lamming G.E. (1995). The oxytocin receptor, luteolysis and the maintenance of pregnancy. J. Reprod. Fertil..

[93] McCracken J.A., Custer E.E., Lamsa J.C. (1999). Luteolysis: A neuroendocrine-mediated event. Physiol. Rev..

[94] Vogt Engeland I., Andresen O., Ropstad E., Kindahl H., Waldeland H., Daskin A., Olav Eik L. (1998). Effect of fungal alkaloids on the development of pregnancy and endocrine foetal-placental function in the goat. Anim. Reprod. Sci..

[95] Paterson J., Forcherio C., Larson B., Samford M., Kerley M. (1995). The effects of fescue toxicosis on beef-cattle productivity. J. Anim. Sci..

[96] Duckett S.K., Andrae J.G., Pratt S.L. (2014). Exposure to ergot alkaloids during gestation reduces fetal growth in sheep. Front. Chem..

[97] Jones K.L., King S.S. (2009). Effect of domperidone supplementation of fescue-fed heifers on plasma and follicular fluid fatty acid composition and oocyte quality. J. Anim. Sci..

[98] Hazeleger N.L., Hill D.J., Stubbing R.B., Walton J.S. (1995). Relationship of morphology and follicular fluid environment of bovine oocytes to their developmental potential *in vitro*. Theriogenology.

[99] Schuenemann G.M., Hockett M.E., Edwards J.L., Tohrbach N.R., Breuel K.F., Schrick F.N. (2005). Embryo development and survival in beef cattle administered ergotamine tartrate to simulate fesecue toxicosis. Reprod. Biol..

[100] Hansen P.J. (2009). Effects of heat stress on mammalian reproduction. Philos. Trans. R. Soc. Lond. B Biol. Sci..

[101] Ealy A.D., Drost M., Hansen P.J. (1993). Developmental changes in embryonic resistance to adverse effects of maternal heat stress in cows. J. Dairy Sci..

[102] Edwards J.L., Hansen P.J. (1997). Differential responses of bovine oocytes and preimplantation embryos to heat shock. Mol. Reprod. Dev..

[103] Brendemuehl J.P., Boosinger T.R., Pugh D.G., Shelby R.A. (1994). Influence of enophyte-infected tall fescue on cyclicity, pregnancy rate and early embryonic loss in the mare. Theriogenology.

[104] Smith S.R., Schwer L., Keene T.C. (2009). Tall Fescue Toxicity for Horses: Literature Review and Kentucky’s Successful Pasture Evaluation Program. Forage Grazinglands.

[105] Putnam M.R., Bransby D.I., Schumacher J., Boosinger T.R., Bush L., Shelby R.A., Vaughan J.T., Ball D., Brendemuehl J.P. (1991). Effects of the fungal endophyte *Acremonium coenophialum* in fescue on pregnant mares and foal viability. Am. J. Vet. Res..

[106] Hovermale J.T., Craig A.M. (2001). Correlation of ergovaline and lolitrem B levels in endophyte-infected perennial ryegrass (*Lolium perenne*). J. Vet. Diagn. Investig..

[107] Youngblood R.C., Filipov N.M., Rude B.J., Christiansen D.L., Hopper R.M., Gerard P.D., Hill N.S., Fitzgerald B.P., Ryan P.L. (2004). Effects of short-term early gestational exposure to endophyte-infected tall fescue diets on plasma 3,4-dihydroxyphenyl acetic acid and fetal development in mares. J. Anim. Sci..

[108] Arns M.J., Pruitt J.A., Sharp C., Wood C., Northcutt S. (1997). Influence of Endophyte-Infected Tall Fescue Seed Consumption on the Establishment and Maintenance of Pregnancy in Mares. Prof. Anim. Sci..

[109] Indänpään-Heikkilä J.E., Schoolar J.C. (1969). LSD: Autoradiographic study of the placental transfer and tissue distribution in mice. Science.

[110] Leist K.H., Grauwiler J. (1973). Transplacental passage of 3H-ergotamine in the rat, and the determination of the intra-amniotic embryotoxicity of ergotamine. Experientia.

[111] Britt J.L., Greene M.A., Bridges W.C., Klotz J.L., Aiken G.E., Andrae J.G., Pratt S.L., Long N.M., Schrick F.N., Strickland J.R. (2019). Ergot alkaloid exposure during gestation alters. I. Maternal characteristics and placental development of pregnant ewes. J. Anim. Sci..

[112] Ehrhardt R.A., Bell A.W. (1995). Growth and metabolism of the ovine placenta during mid-gestation. Placenta.

[113] Borowicz P.P., Arnold D.R., Johnson M.L., Grazul-Bilska A.T., Redmer D.A., Reynolds L.P. (2007). Placental growth throughout the last two thirds of pregnancy in sheep: Vascular development and angiogenic factor expression. Biol. Reprod..

[114] Garrett L.M., Heimann E.D., Wilson L.L., Pfander W.H. (1980). Reproductive problems of pregnant mares grazing fescue pastures. J. Anim. Sci..

[115] Poppenga R.H., Mostrum M.S., Haschek W.M., Lock T.F., Buck W.B., Beasley V.R. Mare agalactia, placental thickening, and high foal mortality associated with the grazing of tall fescue: A care report. Proceedings of the Annual Meeting–American Association of Veterinary Laboratory Diagnosticians.

[116] Monroe J.L., Cross D.L., Hudson L.W., Henricks D.M., Kennedy S.W., Bridges W.C. (1988). Effect of selenium and endophyte-contaminated fescue on performance and reproduction in mares. J. Equine Vet. Sci..

[117] Beers K.W., Piper E.L. (1987). Effect of grazing endophyte-infected fescue on heifer growth, calving rate and calf birth weight, of first calf heifers. Ark. Farm Res..

[118] Watson R.H., McCann M.A., Parish J.A., Hoveland C.S., Thompson F.N., Bouton J.H. (2004). Productivity of cow-calf pairs grazing tall fescue pastures infected with either the wild-type endophyte or a nonergot alkaloid-producing endophyte strain, AR542. J. Anim. Sci..

[119] Antonazzo P., Alvino G., Cozzi V., Grati F.R., Tabano S., Sirchia S., Miozzo M., Cetin I. (2008). Placental IGF2 expression in normal and intrauterine growth restricted (IUGR) pregnancies. Placenta.

[120] Fowden A.L., Sibley C., Reik W., Constanicia M. (2006). Imprinted genes, placental development and fetal growth. Horm. Res..

[121] Akers R.M., Bauman D.E., Capuco A.V., Goodman G.T., Tucker H.A. (1981). Prolactin regulation of milk secretion and biochemical differentiation of mammary epithelial cells in periparturient cows. Endocrinology.

[122] Walner B.M., Booth N.H., Robbins J.D., Bacon C.W., Porter J.K., Kiser T.E., Wilson R., Johnson B. (1983). Effect of an endophytic fungus isolated from toxic pasture grass on serum prolactin concentrations in the lactating cow. Am. J. Vet. Res..

[123] Baldwin R.L., Capuco A.V., Evock-Clover C.M., Grossi P., Choudhary R.K., Vanzant E.S., Elsasser T.H., Bertoni G., Trevisi E., Aiken G.E. (2016). Consumption of endophyte-infected fescue seed during the dry period does not decrease milk production in the following lactation. J Dairy Sci..

[124] Capuco A.V., Bickhart D., Li C., Evock-Clover C.M., Choudhary R.K., Grossi P., Bertoni G., Trevisi E., Aiken G.E., McLeod K.R. (2018). Effect of consuming endophyte-infected fescue seed on transcript abundance in the mammary gland of lactating and dry cows, as assessed by RNA sequencing. J Dairy Sci..

[125] Forsyth I.A. (1986). Variation among species in the endocrine control of mammary growth hormone and placental lactogen. J. Dairy Sci..

[126] Redmond L.M., Cross D.L., Kennedy S.W. (1993). Effect of three levels of domperidone on gravid mares grazing endophyte (*Acremonium coenophialum*) infected tall fescue. J. Anim. Sci..

[127] Altom E.K., Cross D.L., Roach D.K., Strickland J.W., Greene E.M., Clare K.A., Oliver J.W. (1995). The effect of short duration domperidone therapy on gravid mares consuming endophyte infected fescue. J. Anim. Sci..

